# Multi-frequency radiation of dissipative solitons in optical fiber cavities

**DOI:** 10.1038/s41598-020-65426-x

**Published:** 2020-06-01

**Authors:** Oliver Melchert, Ayhan Demircan, Alexey Yulin

**Affiliations:** 1 Institute of Quantum Optics, Leibniz Universität Hannover, Welfengarten 1, 30167 Hannover, Germany; 2 Cluster of Excellence PhoenixD, Welfengarten 1, 30167 Hannover, Germany; 3 Department of Nanophotonics and Metamaterials, ITMO University, 199034 Saint Petersburg, Russia

**Keywords:** Optics and photonics, Mathematics and computing

## Abstract

New resonant emission of dispersive waves by oscillating solitary structures in optical fiber cavities is considered analytically and numerically. The pulse propagation is described in the framework of the Lugiato-Lefever equation when a Hopf-bifurcation can result in the formation of oscillating dissipative solitons. The resonance condition for the radiation of the dissipative oscillating solitons is derived and it is demonstrated that the predicted resonances match the spectral lines observed in numerical simulations perfectly. The complex recoil of the radiation on the soliton dynamics is discussed. The reported effect can have importance for the generation of frequency combs in nonlinear microring resonators.

## Introduction

In recent time resonant radiation of solitons propagating in fibers with high order dispersion attracts much of attention because of both the fundamental interest and practical importance^1,2^. In the pioneering work by Akhmediev and Karlsson^3^ it was discovered that optical solitons propagating in conservative fibers with focusing Kerr nonlinearity and high order dispersion can resonantly emit dispersive waves. The condition of the resonance is the equality of the soliton velocity to the phase velocity of a dispersive wave. This effect is similar to the effect of Cherenkov radiation of charged particles moving at a superluminal velocity. Later it was shown that Bragg solitons moving in a periodic medium emit resonant radiation which is analogous to the transitional radiation of charges flying in a periodic system^4^. Periodical variations of the soliton parameters can lead to resonant radiation, too. It has been shown that resonant radiation can be excited by solitons in periodical systems where the motion of the solitons results in their oscillations. For the case of optical nonlinear Schrödinger solitons this effect was reported in the literature^5,6^ where it was shown that the radiation appears if phase matching conditions are satisfied. It should be mentioned here that the effect of resonant radiation of oscillating solitons is of general nature and can be observed in different physical systems, for example in long Josephson junctions^7^.

The aim of the present paper is to address the resonant radiation of oscillating optical solitons in the presence of high-order dispersion and to reveal the analogy of this radiation to the synchrotron radiation of charges appearing when the velocity of relativistic charges varies periodically in time^8^. It should be noted here that from the point of view of the field theory the synchrotron radiation can be interpreted as an excitation of resonant waves by an oscillating localized source moving at a relativistic velocity. The periodical variations of the velocity of a relativistic charge is just a way to obtain the right driving force in the equation for the electromagnetic waves. Aiming to develop the analogy between synchrotron radiations of the charges and solitons it is much simpler and more instructive to consider one-dimensional oscillating solitons rather than two-dimensional nonlinear localized structures relativistically moving along one direction and wobbling in the transverse direction (though the latter is also possible).

The effects of Cherenkov and synchrotron radiation are widely used in vacuum electronics, in particular in forward- and backward waves oscillators. The optical counterparts of Cherenkov effects can play an important role in the generation of new optical frequencies by the solitons propagating in fibers with high order dispersion. Indeed, since 1995 when Cherenkov radiation of optical solitons was reported for the first time this effect has been actively studied both experimentally and theoretically, especially in the context of the generation of optical supercontinuum spectra^1,2^. Recently, studies with focus on Cherenkov emission shed by shock waves^9^ and ultra-short pulses^10–13^ were published. It was discovered that the effect of Cherenkov radiation affects not only the intensity but also the frequency of the resonantly emitted waves^14,15^.

All these effects are important for the explanation of the dynamics of solitons and domain walls in wave-guiding systems. The effect of synchrotron emission of oscillating solitons can also affect the dynamics of the solitons strongly and thus is of interest from both fundamental and practical points of view. For example, it can possibly be used for the design of optical analogues of gyrotrons and free-electron lasers. Optical solitons in fibers seem to be the best candidates for experimental observation of the discussed effect of synchrotron emission, however similar effects can take place in polariton condensates, plasmas, hydrodynamics and other systems of different physical origins. The resonant emission is possible not only in the conservative but also in dissipative systems providing that the dissipation is not too strong and slowly decaying waves can propagate in the medium. The advantage of weakly dissipative systems is that the resonant radiation can occur there in a stationary regime when it is not disguised by any transitional processes. For this reason, the present study is focused on weakly dissipative annular optical waveguides with high order dispersion, resonantly pumped by external coherent light. Our choice is also motivated by the fact that these systems are not only a convenient test bench for investigation of synchrotron radiation but are of interest on their own and have been actively investigated in recent years theoretically and experimentally. For instance, it was shown that clusters of solitons forming in such systems can be used for information storage and processing^16^. The possibility of frequency comb generation in optic ring microresonators^17–21^ is another motivation to study these systems. The effect of high-order dispersion on the dynamics of the solitons in such systems was investigated and it was shown that high-order dispersion can be crucial for stabilization or destabilization of the soliton clusters^22–28^. The steering of solitary structures by dispersive waves is an important effect discussed in the literature^29–32^. An important problem of the formation of stable bound states of dissipative solitons interacting through the radiation field was considered in detail in terms of a theoretical approach^33^ and the experimental observation of the dispersive waves mediated interaction between solitons in fiber cavities^34^.

Propagation of oscillating solitons in the presence of high order dispersion was studied experimentally and numerically and very rich dynamics of the solitons was reported^35–38^. Cherenkov radiation of oscillating cavity solitons was recently considered^39^ but the effect of the synchrotron radiation was out of the scope of that paper. In the present article we consider synchrotron radiation of oscillating solitons propagating in an optical fiber cavity with third order dispersion.

## Results

To describe the cavity we adopt a well known Lugiato-Lefever model^40^ in the form^27^

1 $${\partial }_{t}A=-\,(1+i\theta )A+i|A{|}^{2}A+i{\partial }_{x}^{2}A+{d}_{3}{\partial }_{x}^{3}A+P,$$

where *θ* is the detuning between the cavity resonance and the frequency of the pump, *P* is the amplitude of the pump and *d*_3_ is the third order dispersion. To make the resonance spectral lines well pronounced we choose the detuning to be equal to a realistic value *θ* = 15, just slightly larger than the value reported by a numerical study reported in the literature^27^. For fiber cavities the boundary conditions are periodic and the spectrum of the eigenmodes is discrete. In this paper we restrict ourselves to the case of cavities so long that the spectrum of the eigenmodes can be considered quasi-continuous. However, we remark that in the case of shorter cavities the boundary conditions can play an important role^41^. In the literature, propagation models represented by equations of the kind of Eq. (1) are used to describe the propagation of optical pulses in media with high-order dispersion and nonlinearity, particularly in microring resonators^27,39^. Equation (1) has bright soliton solutions that loose stability through Andronov-Hopf bifurcation leading to the formation of an oscillating soliton^42,43^ for the pumps exceeding a threshold value. For the chosen parameters the threshold pump is *P*_*th*_ ≈ 7. It is known that introduction of third order dispersion leads to Cherenkov emission of the dispersive waves resulting in a well resolved line in the radiation spectra^39^. Let us now consider the emission of resonant radiation by oscillating dissipative soliton propagating in a cavity with small but non zero third order dispersion. We choose the pump *P* = 8 corresponding to the unstable soliton and wait until instability develops and a stationary oscillating state forms. As initial condition for *t*-propagation in terms of Eq. (1), we use a stationary solution (${\partial }_{t}A(x,t)=0$) of the standard Lugiato-Lefever equation (*d*_3_ = 0 in Eq. (1)). The construction procedure for these initial conditions is detailed in the Supplementary.

### Stationary dynamics of dissipative solitons including third-order dispersion

**Figure 1:**
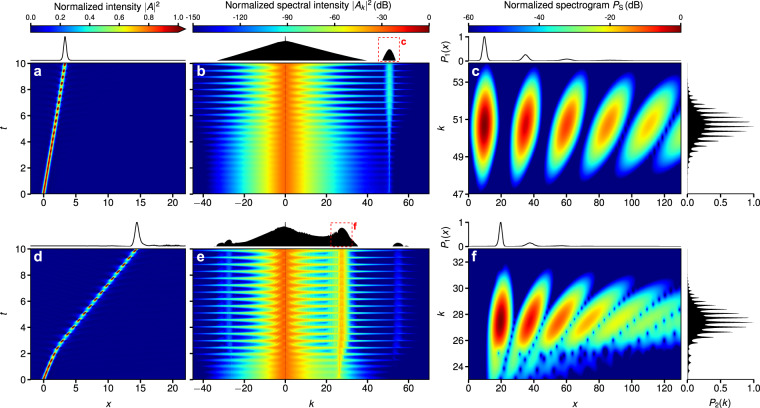
Stationary dynamics of dissipative solitons affected by third-order dispersion. Temporal evolution of (**a**) intensity |*A*(*x*, *t*)|^2^, (**b**) spectral intensity |*A*_*k*_(*t*)|^2^ (dashed box highlights the resonantly generated radiation), and, (**c**) spectrogram *P*_*S*_(*x*, *k*) (see Supplementary for details) for *d*_3_ = 0.02. (**d**–**f**) same for *d*_3_ = 0.04.

Examples of the resulting dynamics for two choices of the third-order dispersion parameter *d*_3_ are shown in Fig. 1. The temporal evolution of the intensity |*A*(*x*, *t*)|^2^ of the field for the small value *d*_3_ = 0.02 is shown in Fig. 1a. It is clearly seen that the soliton oscillates in time. The behavior of the spectrum is shown is Fig. 1b and temporal oscillations of the spectral intensity $S(k,t)=|{A}_{k}(t){|}^{2}$ are also clearly visible. Since the third order dispersion is small, the intensity of the spectral line of the resonant radiation is much weaker compared to the soliton spectrum. The location of the Cherenkov emission at $k\approx 50$, see the dashed box in the top panel of Fig. 1b, is predicted by the resonance condition^39^. A close up view of the frequency range *k* ≈ 47–54 in terms of a spectrogram *P*_*S*_(*x*, *k*) is shown in Fig. 1c (see the Supplementary for details). As evident from the spectrogram, the distinct dispersive waves in the *x* domain exhibit a polychotomous, i.e. multi-peaked, structure in the *k* domain. The intensity of the resonant radiation strongly depends on the strength of high order dispersion and for larger dispersion the radiation spectrum becomes more intense, see Fig. 1d–f for the choice *d*_3_ = 0.04. While the behavior is qualitatively similar, the periodic recoil of the soliton becomes more apparent and the resonant radiation is shifted towards smaller values of *k* to *k* ≈ 28. The positions of the spectral lines observed in numerical experiment match the predictions of the resonant condition if we account for the change of the soliton velocity appearing because of the recoil of the resonant radiation. The dependence of the spectral characteristics of the resonant radiation on the strength of third-order dispersion in the range *d*_3_ = 0.02–0.04 is shown in the Supplementary Information (see Fig. S6a–c).

**Figure 2:**
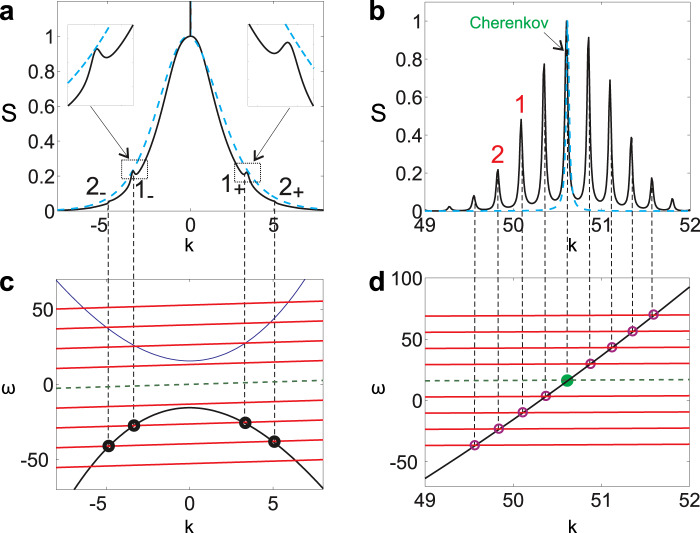
Panel (a) shows the spectrum at low *k* for the oscillating (black solid line) and non-oscillating solitons (blue dashed line). The spectrum in the vicinity of the Cherenkov resonance is shown in panel (b). Graphical solutions of the resonance condition, Eq. (6), are shown in panels (c,d), the green dashed line corresponds to the Cherenkov resonance and the red ones to the resonances with *l* ≠ 0. Resonances marked as 1_+_, 1_−_ on panel (a) and by 1 on panel (b) are given by different crossings of the same red line with the dispersion characteristic of the linear excitations on the soliton background; the same is for the resonances marked by 2. The third order dispersion is *d*_3_ = 0.02, the pump is *P* = 8.

An important fact is that the spectrum of the resonant radiation consist not of a single line as it should be in the case of Cherenkov radiation but of a series of well resolved peaks. Below we will show that, indeed, one of these lines corresponds to Cherenkov radiation whereas the other lines can be refereed to as synchrotron radiation. In Fig. 2a it is also evident that at small *k* there are narrow lines overlapping with the soliton spectrum. These lines can be understood as non-relativistic radiation of the oscillating solitons and thus are analogous to, for example, cyclotron radiation of charged particles. Below we refer to this radiation as cyclotron radiation of solitons, though it is not the only possible analogy. It is worth mentioning here that Eq. (1) is of course not invariant with respect to Lorentz transformation and in this article we use the term “relativistic radiation” only in the sense that the velocity of the soliton is close to the phase velocity of the resonantly generated dispersive waves.

### Resonant radiation of oscillating solitons for small values of *d*_3_

To understand the observed phenomenon let us derive the conditions of resonant radiation of oscillating solitons. Here we only sketch the derivation; the complete analysis will be presented elsewhere. Assuming that the radiation is weak it is possible to look for a solution of the form $A={A}_{0}+u[t,x]$, where *A*_0_ describes the soliton oscillating with period *T* and moving with velocity *v*; the term *u* accounts for the small correction. Here and below the arguments of the functions are enclosed by square brackets.

It is convenient to write an equation for *u* in vector form $\overrightarrow{u}={({\rm{Re}}u,{\rm{Im}}u)}^{T}$, reading

2 $${\partial }_{t}\overrightarrow{u}+\hat{L}\overrightarrow{u}=\overrightarrow{f}.$$

Therein the linear operator $\hat{L}$ has the form

3 $$\hat{L}=(\begin{array}{cc}1+{\rm{Im}}\,{A}_{0}^{2}+{d}_{3}{\partial }_{x}^{3} & 2I-{\rm{Re}}\,{A}_{0}^{2}-\theta +{\partial }_{x}^{2}\\ -\,2I-{\rm{Re}}\,{A}_{0}^{2}+\theta -{\partial }_{x}^{2} & 1-{\rm{Im}}\,{A}_{0}^{2}+{d}_{3}{\partial }_{x}^{3}\end{array}),$$

where $I=|{A}_{0}{|}^{2}$. The right hand side is given by $\overrightarrow{f}={({\rm{Re}}{f}_{0},{\rm{Im}}{f}_{0})}^{T}$ with

4 $${f}_{0}=P-(1+i\theta -i|{A}_{0}{|}^{2}-i{\partial }_{x}^{2}-{d}_{3}{\partial }_{x}^{3}){A}_{0}-{\partial }_{t}{A}_{0}.$$

The left hand side of Eq. (2) describes propagation of waves on the background hosting the soliton. In the conservative case the resonance of the right hand side $\overrightarrow{f}$ with a delocalized eigenmode of the medium results in the formation of a continuous wave propagating away from the soliton. In the dissipative case, of course, the correction *u* is always localized but if the dissipation is weak then the resonant radiation have a form of a slowly decaying wave with the characteristic length depending on the losses and the group velocity of the radiation. The oscillations of the soliton make the coefficients in $\hat{L}$ to be periodic functions of time. It means that the eigenfunctions of the operator $\hat{L}$ are Bloch functions in time and can be sought in the form ${A}_{\omega }={\chi }_{\omega }[t]\cdot {e}^{i\omega t}\cdot {\xi }_{\omega }[x]$ where ${\chi }_{\omega }[t]={\chi }_{\omega }[t\,+\,T]$ are periodic functions of time. The important fact is that far from the soliton the problem becomes uniform in both space and time and thus the asymptotic of the delocalized eigenmodes at $x\to \pm \,\infty$ are just plane waves ${\xi }_{\omega }\sim {e}^{i\omega t-ikx}$ parametrized by the frequency $\omega$ and the wave vector *k*. Resolving the equations for the asymptotic one can easily obtain the dispersion characteristics of the delocalized eigenmodes of the system. This dispersion is, of course, nothing else but the dispersion characteristics of plane waves $\omega ={\omega }_{\pm }[k]$ on the background

5 $${\omega }_{\pm }=i+{d}_{3}{k}^{3}\pm \sqrt{(\theta +{k}^{2}-{I}_{0})\,(\theta +{k}^{2}-3{I}_{0})},$$

where ${I}_{0}=I[\,\pm \,\infty ]$ is the intensity of the background.

To proceed, we use the fact that the soliton oscillates with a period *T* and represents the field in the form $\overrightarrow{f}={\sum }_{l}\,{\overrightarrow{U}}_{l}[x-vt]\cdot {e}^{il{\omega }_{0}t}$ where ${\omega }_{0}=2\pi /T$ is the fundamental frequency of the soliton. The functions *U*_*l*_ are the amplitudes of the temporal Fourier-expansion of the soliton field in the reference frame moving with the soliton. Then each of the terms in the sum can be represented as series over the eigenfunctions of the operator $\hat{L}$. After some straightforward algebra one can establish that the expansion will contain only modes with the frequencies $\omega =kv+l{\omega }_{0}$. The emission of radiation takes place when a harmonic of the right hand side $\overrightarrow{f}$ is in resonance with an eigenwave of the medium. This gives the condition of the phase synchronism

6 $${\rm{Re}}\,[{\omega }_{\pm }({k}_{r})]={k}_{r}v+l{\omega }_{0}.$$

For *l* = 0 this is the condition of Cherenkov radiation. If the condition is satisfied for *l* ≠ 0 then the radiated wave is excited by the *l*-th temporal harmonic of the oscillating soliton. Let us now discuss how the derived resonance conditions explain the results of the numerical experiments. To do this we study the spectrum and the resonance conditions at low *k* and at *k* close to Cherenkov resonance. We start with the resonances at small wave vectors *k*. The spectrum obtained in numerical simulations is shown in panel (a) of Fig. 2. The spectrum looks like a bell-shaped function with the narrow line at *k* = 0 corresponding to the background. Comparing spectra of non-oscillating and oscillating solitons one can notice that in the latter case several additional maxima appear on the spectral curve.

**Figure 3:**
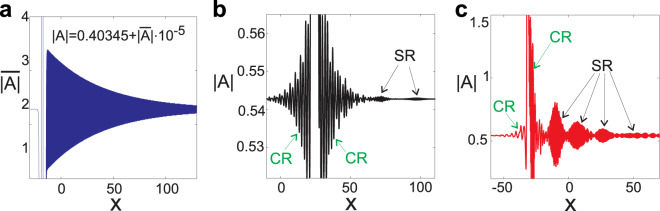
(**a**) Radiation field of a non-oscillating soliton for parameter values *P* = 6, and *d*_3_ = 0.02. (**b**) Radiation of an oscillating soliton for *P* = 8 and *d*_3_ = 0.02. (**c**) same for *P* = 8 and *d*_3_ = 0.06. In panels (b,c) synchrotron radiation is labeled “SR” and cyclotron radiation is labeled “CR”.

To explain the observed maxima on the spectrum we solve the resonance conditions given by Eq. (6) graphically. These solutions for −4 ≤ *l* ≤ 4 are shown in Fig. 2c. The soliton velocity used in the resonance conditions was extracted from numerical simulations. It is seen that the green line [corresponding to *l*  = 0 in Eq. (6)] does not cross the dispersion characteristics of linear waves and so Cherenkov radiation do not occur at low *k*. In our case the resonance conditions can not be satisfied for *l* = ±1 either but for larger |*l*| ≥ 2 the resonance condition is met. Comparing the positions of the crossings with the positions of the small maxima on the spectrum we see that they match perfectly. The phase velocity of these resonant modes are much higher comparing to the soliton velocity and thus developing the analogy between the radiation of solitons and moving charges we can refer these resonances as cyclotron radiation of solitons. Let us note that the emitted waves with *k* < 0 propagate with the group velocity greater than the velocity of the soliton and so the radiation appears in front of the soliton. The emitted radiation with *k* > 0 propagates in the opposite direction and so it always appears behind the soliton. Topologically it is obvious that if there is a resonance at a positive *k* then there is a resonance at a negative *k* and so cyclotron radiation always appears simultaneously in front and behind the soliton, see Fig. 3b,c and Supplementary Video SV1, showing the field distributions of oscillating solitons. Now let us consider the radiation with the frequencies close to the Cherenkov resonance. The spectrum of the stationary field obtained in numerical simulations and graphical solutions of the resonance conditions are shown in Fig. 2b,d, correspondingly. The crossing of the green dashed line (*l* = 0) with the dispersion characteristics gives the position of Cherenkov resonance and it is seen that it matches the position of the one of the spectral lines observed in numerical experiment perfectly. The other resonances (*l* ≠ 0) fit the other spectral lines. So we can explain the spectral structure of soliton radiation as a number of lines produced by different temporal harmonics of the oscillating soliton. It is obvious that the emitted waves have phase velocities close to the velocity of the soliton (for Cherenkov resonance they are exactly equal) and thus these radiations can be interpreted as relativistic radiation of the oscillating soliton, i.e. synchrotron radiation. Looking at the dispersion characteristic in the vicinity of the resonances one can conclude that in the considered case Cherenkov and synchrotron radiations always have group velocities higher than the velocity of the soliton and so the radiation must appear in front of the soliton. It is instructive to compare the radiation of oscillating and not oscillating solitons. The blue dashed curves in Fig. 2a,b show the spectrum of a non oscillating soliton forming in the case of a weaker pump *P* = 6. The velocities of the solitons are practically the same for *P* = 6 and *P* = 8 and so the Cherenkov spectral lines of the oscillating and non-oscillating solitons practically coincide. However it is clearly seen that the non-oscillating soliton emits neither cyclotron no synchrotron radiation. In the case of third order dispersion the synchrotron resonances are always situated on the left and on the right of the Cherenkov spectral line, but it is important to remark here that there are dispersion characteristics allowing for synchrotron resonances for *l* ≠ 0 in the absence of the Cherenkov one.

To shed more light on the resonant emission we discuss the shapes of the radiation fields. Figure 3a shows stationary Cherenkov radiation of a non-oscillating soliton pumped at *P* = 6 when third order dispersion is set to *d*_3_ = 0.02. The resonantly emitted radiation contains only a single mode, see the blue dashed line in Fig. 2b and decays exponentially with the rate 1/[*v*_*g*_ − *v*]. An account of the temporal evolution of the corresponding intensity, spectral intensity as well as a spectrogram at propagation time *t* = 10 is given in the Supplementary. For stronger pump *P* = 8 the soliton develops oscillations and, as discussed above, the cyclotron radiation appears in front and behind the soliton. This radiation is clearly seen in Fig. 3b. The radiation at low *k* has small group velocity and decays quite quickly in *x*. Consequently, this radiation is seen only in the immediate vicinity of the soliton. Cherenkov and synchrotron radiation at large *k* have higher group velocities and thus decay slower in *x* than the radiation at low *k*. In Fig. 3b this radiation (labeled SR) is weak but visible at *x* ≥ 60 (cf. Fig. 1c). In the case of stronger third order dispersion *d*_3_ = 0.06 the synchrotron radiation is much more pronounced and clearly seen in Fig. 3c (cf. Fig. 1f). The increase of third-order dispersion implies that the zero-dispersion frequency shifts closer to the soliton.

A very important fact is that the synchrotron radiation of an oscillating soliton is pulsating (see Supplementary Video SV1) whereas Cherenkov radiation of a non-oscillating soliton is a slowly decaying continuous wave (see Supplementary Video SV2). The effect of the pulsation can be explained by the interference of synchrotron emissions at close frequencies. The distances between the synchrotron resonances can be estimated as Δ$k={\omega }_{0}/[{v}_{gs}-v]$ and Δ$\omega =\frac{|{v}_{gs}|}{|{v}_{gs}-v|}{\omega }_{0}$ where ${v}_{gs}={\partial }_{k}\omega ({k}_{r})$ is the group velocity of the radiation. Then the length of the radiation pulses is 2*πv*_*gs*_/Δ$\omega$ giving ≈15 at *d*_3_ = 0.06 which fits well to the lengths of the pulses observed in our numerical simulations.

### Resonant radiation of oscillating solitons for large values of *d*_3_

**Figure 4:**
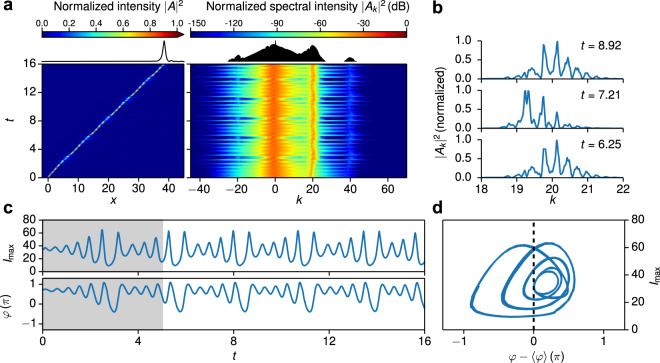
Dynamics of an oscillating soliton at *d*_3_ = 0.06. (**a**) Temporal evolution of the intensity (left), and spectral intensity (right) for *d*_3_ = 0.06. (**b**) Close up view of the normalized spectrum in the range *k* = 18–22, i.e. the synchrotron part of the spectrum, at selected propagation times. (**c**) Dynamics in terms of the maximal intensity *I*_max_(*t*) (top) and phase *φ*(*t*) (bottom). The shaded area indicates an initial transient phase in the range *t* < 5. (**d**) Dynamics of the soliton in the $({I}_{{\rm{\max }}},\varphi -\langle \varphi \rangle )$–plane, restricted to *t* > 5. The dashed line indicates the Poincaré section **d**efined by $\varphi (t)=\langle \varphi \rangle$.

Above we considered mostly the case of small third order dispersion when the resonant emission is very weak. Now we turn our attention to the case of stronger high order dispersion when a recoil from the radiation becomes important, see Fig. 4a. Subsequently, we address the recoil from the radiation on the oscillating soliton. From our numerical simulations we extract the maximum intensity ${I}_{{\rm{\max }}}[t]=|A[x{\prime} ,t]{|}^{2}$ upon propagation in time and determine the corresponding phase $\varphi [t]={\tan }^{-1}\,\{{\rm{Im}}\,(A[x{\prime} ,t])/{\rm{Re}}(A[x{\prime} ,t])\}$ at $x{\prime} ={\rm{\arg }}\,{{\rm{\max }}}_{x}\,|A[x,t]|$. An example for the dynamics of an oscillating soliton at *d*_3_ = 0.06 is shown in Fig. 4c. Both, the maximum intensity and phase, parametrically define curves in a plane spanned by the variables $({q}_{1}[t],{q}_{2}[t])=({I}_{{\rm{\max }}}[t],\varphi [t]-\langle \varphi \rangle )$. Neglecting the initial transient behavior observed for *t* < 5, the trajectory resulting at *d*_3_ = 0.06 reveals a limit-cycle of period 5, see Fig. 4d. The time it takes the system to traverse the limit cycle is *t*_0_ ≈ 2.7. As evident from the close up view of the synchrotron part of the spectrum in Fig. 4b, the intensities and peak-positions vary in time but recur after the temporal period *t*_0_.

**Figure 5:**
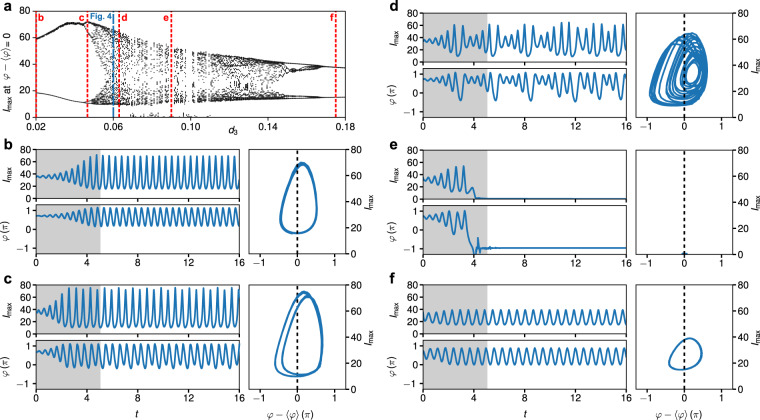
Dynamics of oscillating solitons in the parameter range *d*_3_ = 0.02 through 0.18. (**a**) Bifurcation diagram summarizing different regimes of motion in the range *d*_3_ = 0.02–0.18. (**b**) Dynamics for *d*_3_ = 0.02. The left panel shows the maximal intensity *I*_max_ (top) and phase *φ* (bottom), with the shaded area indicating an initial transient phase for *t* < 5. The right panel shows the dynamics of the soliton in the $({I}_{{\rm{\max }}},\varphi -\langle \varphi \rangle )$–plane, restricted to *t* > 5, with the dashed line indicating the section defined by $\varphi (t)=\langle \varphi \rangle$. (**c**) Same for *d*_3_ = 0.047, (**d**) Same for *d*_3_ = 0.063, (**e**) Same for *d*_3_ = 0.090, (**f**) Same for *d*_3_ = 0.175.

To summarize the propagation dynamics of oscillating solitons in the parameter range *d*_3_ = 0.02 through 0.18, we show a bifurcation diagram in Fig. 5a. This portrays the structural change of the distrete-time trajectory $({q}_{1}[t],{q}_{2}[t])$ as function of third-order dispersion by computing Poincaré return maps^44^ for a sequence of parameter values *d*_3_. Therefore, at each value of *d*_3_, we record the coordinates of the trajectory whenever it crosses a Poincaré section defined by the line *q*_2_ = 0. To accurately determine the value of *q*_1_ whenever *q*_2_ = 0, we adopt Henons extrapolation-free method^44,45^. The resulting sequence of points ${q}_{1,n},\,n=1\ldots N$, is then registered in the bifurcation diagram at its respective value of *d*_3_. In Fig. 5b–f we illustrate the system dynamics for selected values of *d*_3_ that exhibit a distinguished steady state behavior. The temporal evolution of the intensity and spectral intensity for these cases is shown in the Supplementary Information (Fig. S5). In case of *d*_3_ = 0.02 (Fig. 5b), these points appear clustered at the two values *q*_1,1_ ≈ 67 and *q*_1,2_ ≈ 16, showing that the trajectory alternates between two attractors. The underlying trajectory in the (*q*_1_, *q*_2_)–plane is referred to as a 2-cycle^44^. This comprises the simplest periodic motion in which the soliton periodically oscillates between a unique intensity minimum and maximum. Such a behavior is given at very small values of third order dispersion *d*_3_ < 0.044. For increasing values of *d*_3_ the system passes through a sequence of period doublings. E.g., for *d*_3_ = 0.047 (Fig. 5c), the trajectory alternates between four attractors, yielding a 4-cycle. For *d*_3_ = 0.06 (Fig. 4c,d), the trajectory forms a 10-cycle. No discernible regularity up to *t* = 16 is found for *d*_3_ = 0.063 (Fig. 5d). Here, the long-time behavior of the system is not predictable reminiscent of chaotic dynamics. In the parameter range *d*_3_ = 0.07–0.11 the homogeneous stationary state can become the only stable solution, portraying an extinction event in which the oscillating soliton can vanish completely. An example of this behavior is shown in Fig. 5e for *d*_3_ = 0.09. For larger values of *d*_3_, remerging bifurcation events occur^46^. E.g., at *d*_3_ = 0.175 (Fig. 5f) the trajectory forms again a 2-cycle. For this second region of 2-cycle dynamics, the dependence of the spectral characteristics of the resonant radiation on the strength of third-order dispersion is again shown in the Supplementary Information (see Fig. S6d–f).

Overall, this behavior can be explained by the fact that the pulsations of the synchrotron radiation causes a pulsating recoil of the radiation on the soliton. Such a recoil leads to the oscillating variations of the soliton velocity and intensity. In its turn, the change of the soliton parameters affects the instantaneous frequencies of the radiation. The interplay of these effects can result in a very complex dynamics of the soliton. As evident from Fig. 5a, the complex dynamics of oscillating solitons is observed within an intermediate range of the third-order dispersion coefficient *d*_3_ ≈ 0.44–0.17, whereas nearly periodic oscillations are observed outside this intermediate range. Possibly this explains the complicated dynamics of cavity solitons discussed in the literature^35^.

## Summary

In summary, it is shown that oscillating solitons can emit resonant radiation analogous to the synchrotron and cyclotron radiations of moving charges. The resonance condition for the radiation is derived and it is demonstrated that the predicted resonances match the observed spectral lines very precisely. The structure of the field of the emitted radiation and the effect of the recoil of the radiation on the soliton dynamics were also addressed in the presented study. The reported results are of very general physical nature and thus can be observed not only in fiber cavities but in a wide class of physical systems supporting oscillating solitons.

## Supplementary information

Supplementary Information.

Supplementary Information2.
