# Supplementary material for: Multi-frequency radiation of dissipative solitons in optical fiber cavities
Source: Sci Rep. 2020 Jun 1;10:8849. doi: 10.1038/s41598-020-65426-x (PMC7264339; doi:10.1038/s41598-020-65426-x)
Supplement: Supplementary file 1 — Supplementary Information. [file 41598_2020_65426_MOESM1_ESM.pdf]

Multi-frequency radiation of dissipative solitons in optical fiber cavities (Supplementary Information)

Oliver Melchert,[1,2] Ayhan Demircan,[1,2] and Alexey Yulin[3,2]

[1] *Institute of Quantum Optics, Leibniz Universität Hannover, Welfengarten 1, 30167, Hannover, Germany*
[2] *Cluster of Excellence PhoenixD, Welfengarten 1, 30167, Hannover, Germany*
[3] *Department of Nanophotonics and Metamaterials, ITMO University, Birzhevaya Liniya V.O., 14, 199034, Saint Petersburg, Russia*

CONTENTS

S-1. Details on our numerical simulations 1

S-2. Construction of initial conditions for Eq. (1) 1

S-3. Construction of the spectrograms in Figs. 1(c,f) 2

S-4. Description of the supplementary video 2

S-5. Supporting information for Fig. 3(a) 3

S-6. Supporting information for Fig. 5 3

S-7. Dependence of the resonant radiation on d_3 3

References 3

S-1. DETAILS ON OUR NUMERICAL SIMULATIONS

For the propagation of an initial field $A_0(x)$ in terms of Eq. (1) in the main text we use numerical methods that rely on (i) a pseudospectral scheme implementing t–propagation using a fourth-order Runge-Kutta method, and, (ii) a pseudospectral scheme implementing t–propagation using a split-step approach. In our simulations, the x–domain was bounded by $-160 \leq x < 160$ and discretized using $N_x = 2^{13}$ equidistant mesh-points. The temporal evolution the fields $A(x,t)$ was followed up to a maximal propagation time $t = 16$ using at least $N_t = 4 \times 10^4$ equidistant steps.

To verify reliability of our findings we repeated a subset of the numerical simulations for cavities of different size and for different numbers of discretization points in both, x and t.

S-2. CONSTRUCTION OF INITIAL CONDITIONS FOR EQ. (1)

The initial conditions used for t-propagation in terms of Eq. (1) in the main text are obtained by an analysis of the corresponding standard Lugiato-Lefever equation (LLE). The standard LLE, obtained by setting $d_3 = 0$ in Eq. (1), exhibits field solutions that describe localized dissipative states satisfying $\partial_t A(x,t) = 0$ for a wide range of control parameter values, including the particular choice $\theta = 15$ and $P = 8$ considered in our study.

Here we compute such initial conditions by finding a complex-valued solution $A_0(x)$ such that $F(A_0(x)) = 0$ with

$$F(A) = -(1+i\theta)A + i|A|^2A + i\partial_x^2 A + P. \qquad \text{(S1)}$$

As ansatz for an approximate trial solution for this root-finding problem we take

$$A_{\rm trial}(x) = A_{\rm HSS} + A_{\rm LDS}(x), \qquad \text{(S2)}$$

wherein $A_{\rm HSS}$ denotes a homogeneous steady-state solution (HSS), and where $A_{\rm LDS}$ is a localized dissipative structure (LDS).

The homogeneous steady-state solution of the standard LLE for given control parameters θ and P reads [1]

$$\mathsf{Re}[A_{\rm HSS}] = P/[1+(I_{\rm HSS}-\theta)^2], \qquad \text{(S3)}$$

$$\mathsf{Im}[A_{\rm HSS}] = (I_{\rm HSS}-\theta)P/[1+(I_{\rm HSS}-\theta)^2], \qquad \text{(S4)}$$

with $I_{\rm HSS} = |A_{\rm HSS}|^2$ being a solution of

$$I_{\rm HSS}[1+(\theta - I_{\rm HSS})^2] = P^2, \qquad \text{(S5)}$$

obtained by setting all derivatives of the LLE to zero [2].

As an approximate trial function for a possible localized dispersive structure, the similarity of the LLE to a perturbed nonlinear Schrödinger equation suggests [3, 4]

$$A_{\rm LDS}(x) = \sqrt{2\theta}\,\mathrm{sech}\left(\sqrt{\theta}x\right)\,e^{i\zeta}, \qquad \text{(S6)}$$

resembling a standard nonlinear-Schrödinger soliton. In the considered case, i.e. for a constant control parameter P, the constant phase $\zeta = \arccos[\sqrt{8\theta}/\pi P]$ is found as a consequence of the conservation law $\frac{d}{dt}\int |A_{\rm LDS}(x)|^2\,dx = 0$ under propagation with the standard LLE [4].

For the control parameter ranges considered in our numerical simulation we found that a trial solution given by Eq. (S2), with $A_{\rm HSS}$ and $A_{\rm LDS}(x)$ as detailed above, is already close to a proper stationary solution. Consequently, a root-finding procedure for Eq. (S1), implemented via standard numerical tools, converges quite fast. In Fig. S1 we demonstrate that the stationary solution used as initial condition for the LLE with nonzero

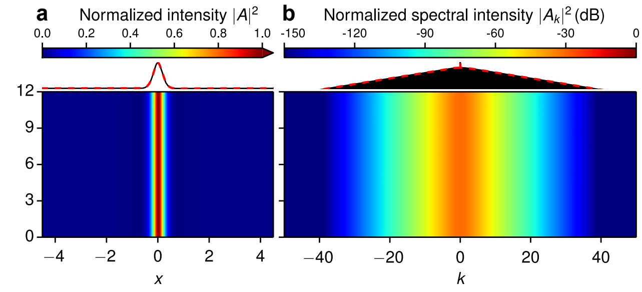

FIG. S1. Demonstration of the stationarity of the initial condition under propagation with the standard LLE. (a) Evolution of the intensity $|A(x,t)|^2$. The black solid (red dashed) line indicates the intensity at $t = 0$ ($t = 12$). (b) Evolution of the spectral intensity $|A_k(t)|^2$. The shaded area (red dashed line) indicates the spectral intensity at $t = 0$ ($t = 12$).

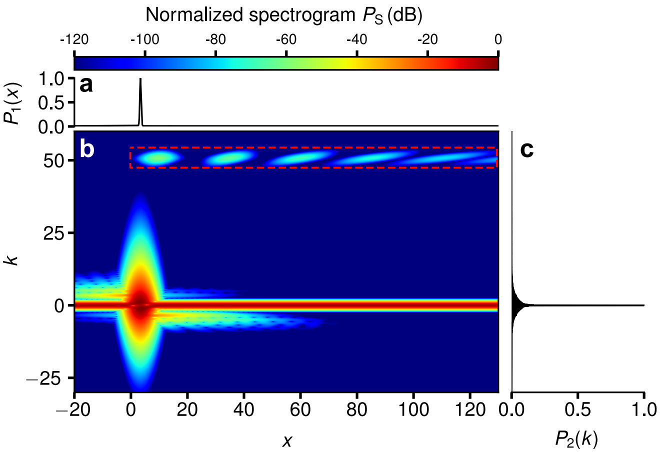

FIG. S2. Spectrogram $P_S(x,k)$ of the field $A(x,t)$ at propagation distance $t = 10$ for control parameter values $d_3 = 0.02$, $\theta = 15$, and $P = 8$. The spectrogram is restricted to the range $(x_{\rm min}, x_{\rm max}) = (-20, 130)$ and $(k_{\rm min}, k_{\rm max}) = (-30, 60)$. (a) x-domain intensity $P_1(x)$ restricted to modes in the range $(k_{\rm min}, k_{\rm max})$. (b) Spectrogram $P_S(x,k)$ calculated using a rms-width $\sigma = 2$. A close up view of the region enclosed by the red dashed line is shown in Fig. 1(c) in the main text. (c) k-domain intensity $P_2(x)$ restricted to the field in the range $(x_{\rm min}, x_{\rm max})$.

d_3 is indeed a stationary solution of the standard LLE. In case the root-finding problem is not solved with sufficient accuracy, the resulting putative stationary solution would soon evolve into an oscillating solution even under the standard LLE.

S-3. CONSTRUCTION OF THE SPECTROGRAMS IN FIGS. 1(C,F)

To unravel the x and k relationships within the complex valued field $A(x,t)$ at a given propagation time t we use a spectrogram [5, 6], as illustrated in Figs. 1(c,f) in the main text. To calculate a spectrogram we here consider a Gaussian window function $h(x) = \exp(-x^2/2\sigma^2)$ to localize the field in the coordinate x. The Fourier transform of the localized field

$$S_x(k) = \frac{1}{\sqrt{2\pi}} \int A(x')h(x'-x)e^{-ikx'}\,{\rm d}x', \qquad \text{(S7)}$$

then provides a spectrogram $P_S(x,k) = |S_x(k)|^2$ by sweeping over x. The choice of the root-mean-square (rms) width σ naturally limits the resolution in x and k, achieved by the spectrogram [5].

An exemplary spectrogram for the field $A(x,t)$ at propagation distance $t = 10$ for control parameter values $d_3 = 0.02$, $\theta = 15$, and $P = 8$ is shown in Fig. S2(b). The spectrogram is restricted to the range $(x_{\rm min}, x_{\rm max}) = (-20, 130)$ and $(k_{\rm min}, k_{\rm max}) = (-30, 60)$. The frequency resolution of the pump at $k = 0$ is due to the finite rms-with $\sigma = 2$ of the Gaussian window function used for field-localization. The figure further shows the dissipative soliton at $x \approx 3$ and the resonantly generated radiation at large values of k, i.e. the synchrotron part of the spectrum, enclosed by a red dashed box. The cyclotron part of the radiation at small values of $|k|$ is also well visible in the spectrogram.

In Fig. S2(a) we show the normalized intensity $P_1(x) = I(x)/\max[I(x)]$ with $I(x) = |\int_{k_{\rm min}}^{k_{\rm max}} A_k e^{-ikx}\,{\rm d}k|^2$. Evolution of the field in terms of intensity and spectral intensity up to $t = 10$ is shown in Figs. 1(a,b) of the main text. The restriction to frequency components in range $(k_{\rm min}, k_{\rm max})$ allows to emphasize features of the intensity that might otherwise be obscured by features that are present in the full field $A(x,t)$ at the selected value of t. This becomes evident by inspection of $P_1(x)$ in Fig. 1(c) in the main text, which allows to clearly distinguish the resonantly generated radiation with modes in the range $(k_{\rm min}, k_{\rm max}) = (47, 54)$. In Fig. S2(a) these are immersed on a constant background contributed by the pump, and further dominated by the intensity of the dissipative soliton at $x \approx 3$.

In Fig. S2(c) we show the normalized intensity $P_2(k) = I_k/\max[I_k]$ with $I_k = |A_k|^2$ restricted to the range $(k_{\rm min}, k_{\rm max})$. While this emphasizes the constant pump in Fig. S2(c), it reveals the small-scale polychotomous structure of the resonantly generated radiation for $(k_{\rm min}, k_{\rm max}) = (47, 54)$ in Fig. 1(c) of the main text.

S-4. DESCRIPTION OF THE SUPPLEMENTARY VIDEO

As supplementeray material we also provide a supplementary video (see supplementary video SV1), detailing the propagation dynamics of an oscillating soliton for controll parameters $\theta = 15$, $P = 8$, and $d_3 = 0.02$, in the propagation range $t = 5-8$. Figure S3 provides a still selected from the supplementary video and provides

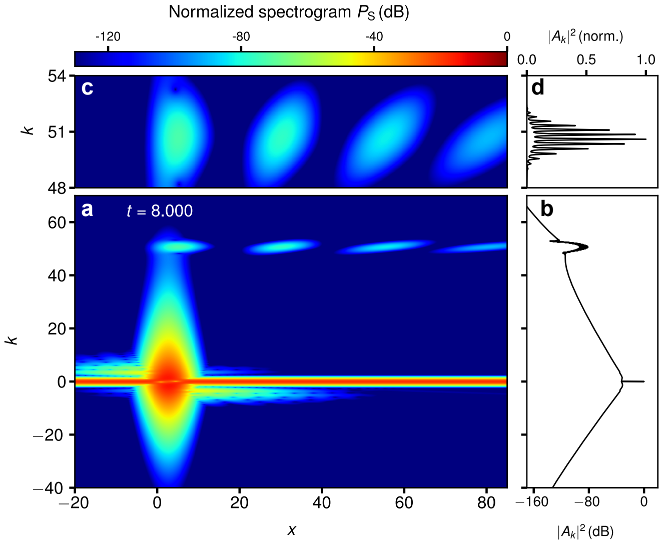

FIG. S3. Still taken from the supplementary video SV1, detailing the propagation dynamics of an oscillating soliton for parameter values $\theta = 15$, $P = 8$, and $d_3 = 0.02$. The still is taken at propagation distance $t = 8$. (a) Spectrogram of the field $A(x, t = 8)$, constructed using a rms-width $\sigma = 2$ of the Gaussian window function. (b) Spectral intensity $|A_k(t = 8)|^2$ on a semi-logarithmic scale, measured in dB relative to the spectral intensity at $k = 0$, $t = 0$. (c) Close up view of the spectrogram in the range $k = 48-54$ where resonant radiation at large values of k, i.e. the synchrotron part of the radiation, is generated. (d) Spectral intensity in the range $k = 48-54$, shown on a linear scale and normalized to the maximum value in that range at the current value of t.

a caption detailing the different panels of the video.

S-5. SUPPORTING INFORMATION FOR FIG. 3(A)

As discussed in the main text, Fig. 3(a) of the main text shows the radiation field of a non-oscillating soliton for the parameter values $P = 6$ and $d_3 = 0.02$. For completeness, in Fig. S4 we show the temporal evolution of the intensity [Fig. S4(a)], spectral intensity [Fig. S4(b)], and a close up view of the resonantly generated radiation in terms of a spectrogram [Fig. S4(c)]. As evident from the spectrogram, the Cherenkov radiation of a non-oscillating soliton is a slowly decaying dispersive wave.

S-6. SUPPORTING INFORMATION FOR FIG. 5

In Fig. 5 in the main text we detailed the dynamics of oscillating solitons for selected values of third-order dispersion in the range $d_3 = 0.02$ through 0.18. Therein, the qualitative change in their dynamics was illustrated using the temporal evolution of the peak intensity $I_{\rm max}$ and phase φ. Here, for completeness, we show the corresponding temporal evolution of the intensity and spectral intensities in Fig. S5.

S-7. DEPENDENCE OF THE RESONANT RADIATION ON d_3

We studied the peak amplitude $I^{\rm RR}_{\rm max} = \max(I^{\rm RR}_k)$ of the resonant radiation and its spectral width, for which we here use the definition of the root-mean squared width

$$w_k = \left[\frac{\int (k - k_{\rm max})^2 I^{\rm RR}_k \, {\rm d}k}{\int I^{\rm RR}_k \, {\rm d}k} \right]^{1/2}, \qquad \text{(S8)}$$

in more detail. In our analysis we focused on two regions of third order dispersion. These are the range of small values $d_3 = 0.02 \ldots 0.04$ (see Fig. S6a-c), and, the range of large values $d_3 = 0.18 \ldots 0.195$ (see Fig. S6d-f). As evident from Fig. 5(a) of the main document, in both these ranges the peak intensity of the oscillating soliton alternates between a unique minimum and maximum. Above, $I^{\rm RR}_{\rm max}$ refers to the spectral intensity of the dominant pulse of resonant radiation, taken at a point in the steady-state regime where the amplitude of the temporal oscillation of the soliton assumes its minimum value. At that point, the soliton is spectrally narrow and the spectral width of the dominant pulse of resonant radiation can be well distinguished. For an example, see the spectrogram in Fig. 1c of the main document, featuring the dominant pulse of resonant radiation within the range $[k = 47 \ldots 54] \times [x = 0 \ldots 20]$. Further, $k_{\rm max}$ in Eq. (S8) refers to the peak position of the dominant pulse in the k-domain. As an example, for the dominant pulse at $d_3 = 0.02$ featured in Fig. 1c, we find $k_{\rm max} = 50.7$ and $w_k = 0.50$, see Fig. S6a-c. At such small values of d_3 we observe that the dominant pulse exhibits a Gaussian profile with full width at half maximum (FWHM) given by $\text{FWHM} \approx 2.35 \cdot w_k$. However, already for $d_3 > 0.03$, the dominant pulse is not well approximated by a Gaussian profile. For a third-order dispersion strength in the range $d_3 = 0.180 - 0.195$ we observe a clear linear increase of the peak amplitude $I^{\rm RR}_{\rm max} = -0.0004 + 0.0058 \cdot d_3$, with accompanying decrease of w_k. For the analysis we have neglected the range of intermediate third-order dispersion strength where the soliton dynamics is rather complex and results in resonant radiation with spectra that strongly vary in time.

[1] P. Parra-Rivas, D. Gomila, P. Colet, and L. Gelens. Interaction of solitons and the formation of bound states in the generalized Lugiato-Lefever equation. *Eur. Phys. J. D*, 71:198, 2017.

[2] P. Parra-Rivas, D. Gomila, M. A. Matías, S. Coen, and L. Gelens. Dynamics of localized and patterned structures in the Lugiato-Lefever equation determine the stability and shape of optical frequency combs. *Phys. Rev. A*, 89:043813, 2014.

[3] J. K. Jang, M. Erkintalo, K. Luo, G.-L. Oppo, S. Coen, and S. G. Murdoch. Controlled merging and annihilation of localised dissipative structures in an AC-driven damped nonlinear Schrödinger system. *New Journal of Physics*, 18:033034, 2016.

[4] W. B. Cardoso, L. Salasnich, and B. A. Malomed. Localized solutions of Lugiato-Lefever equations with focused pump. *Sci. Rep.*, 7:16876, 2017.

[5] L. Cohen. Time-Frequency Distributions – A Review. *Procedings of the IEEE*, 77:941–981, 1989.

[6] O. Melchert, B. Roth, U. Morgner, and A. Demircan. OptFROG – Analytic signal spectrograms with optimized time-frequency resolution. *SoftwareX*, 10:100275, 2019.

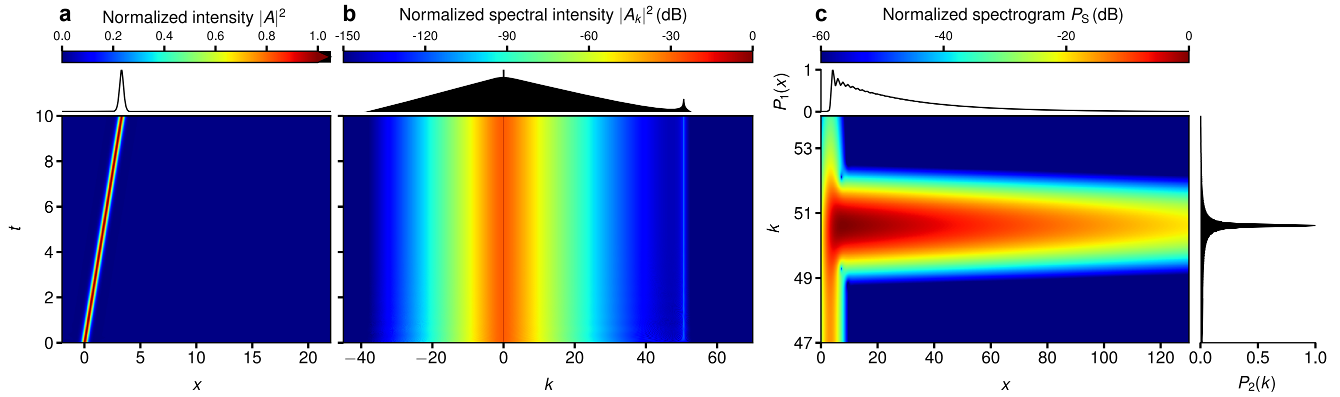

FIG. S4. Stationary dynamics of a non-osscillating dissipative soliton for parameter values $P = 6$ and $d_3 = 0.02$. Temporal evolution of (a) intensity $|A(x,t)|^2$, and, (b) spectral intensity $|A_k(t)|^2$. (c) Spectrogram $P_S(x,k)$ computed using a rms-width $\sigma = 2$.

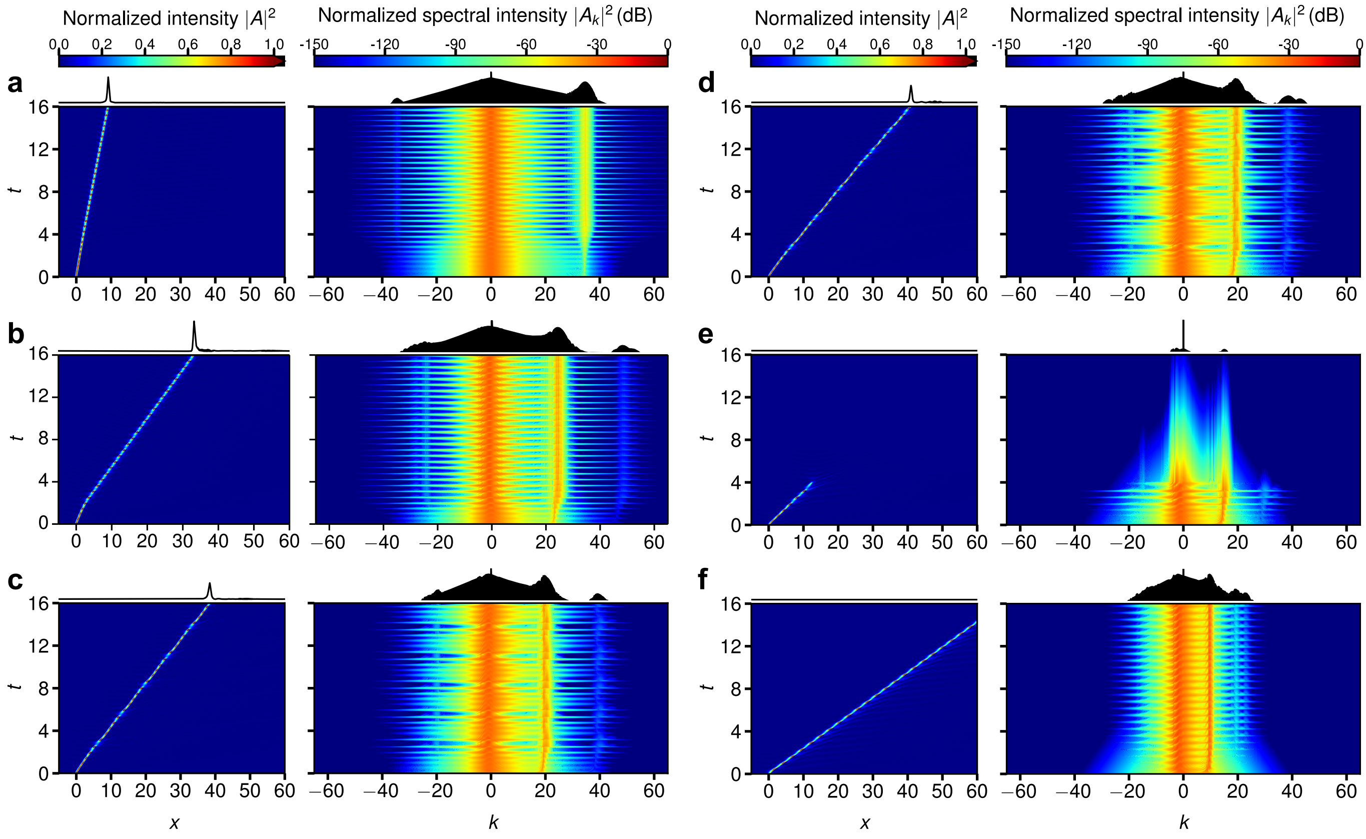

FIG. S5. Distinguished propagation dynamics observed at selected values of d_3 for fixed values $\theta = 15$, and $P = 8$. (a) Temporal evolution of the intensity $|A(x,t)|^2$ (left panel) and spectral intensity $|A_k(t)|^2$ (right panel) for $d_3 = 0.02$, (b) same for $d_3 = 0.047$, (c) same for $d_3 = 0.06$, (d) same for $d_3 = 0.063$, (e) same for $d_3 = 0.09$, and, (f) same for $d_3 = 0.175$.

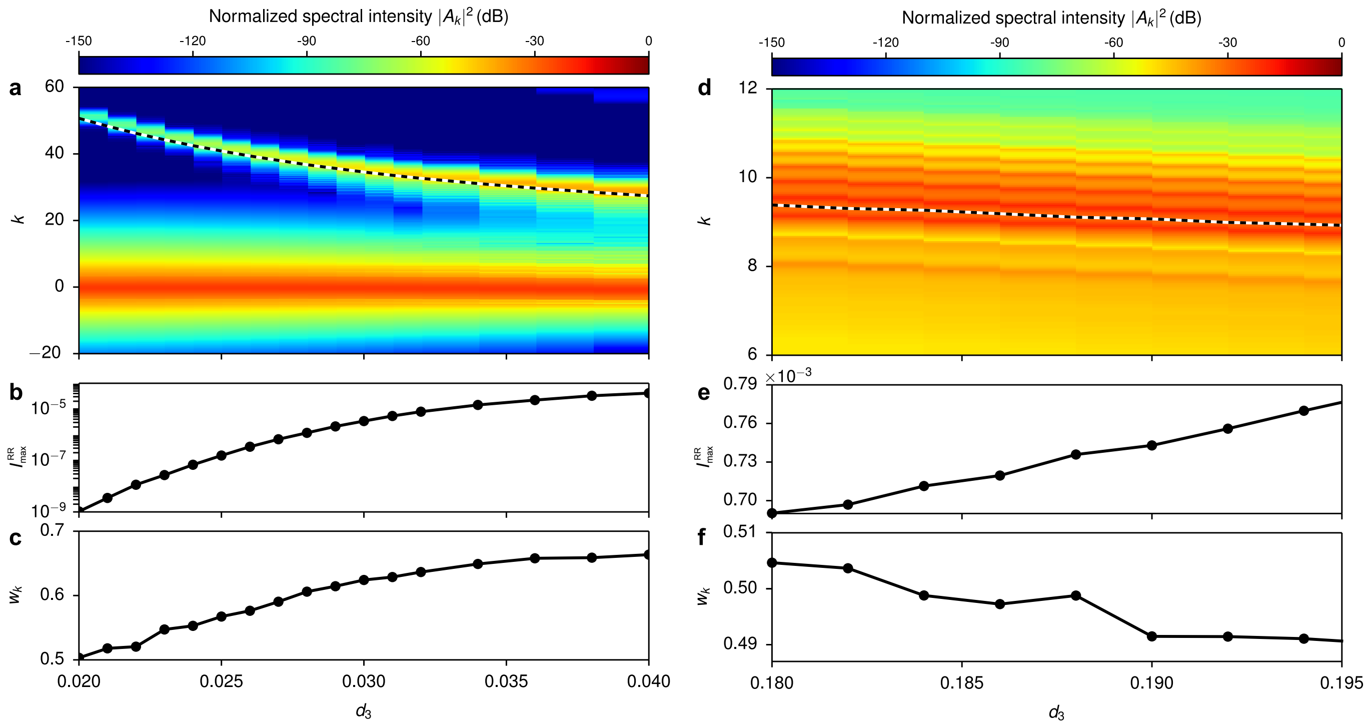

FIG. S6. Dependence of the radiation spectra on d_3. (a) Spectral intensiy and location $k_{\max}$ of the dominant pulse of resonant radiation (dashed line) in the range $d_3 = 0.02 \ldots 0.04$. (b) Increase of the resonant radiation maximum intensity $I^{\mathrm{RR}}_{\max}$, and, (c) increase of the root-mean-sqare width w_k upon increasing d_3. (d-f) same for the region $d_3 = 0.18 \ldots 0.195$.
