# Supplementary figures and images for: Multi-frequency radiation of dissipative solitons in optical fiber cavities
Source: Sci Rep. 2020 Jun 1;10:8849. doi: 10.1038/s41598-020-65426-x (PMC7264339; doi:10.1038/s41598-020-65426-x)

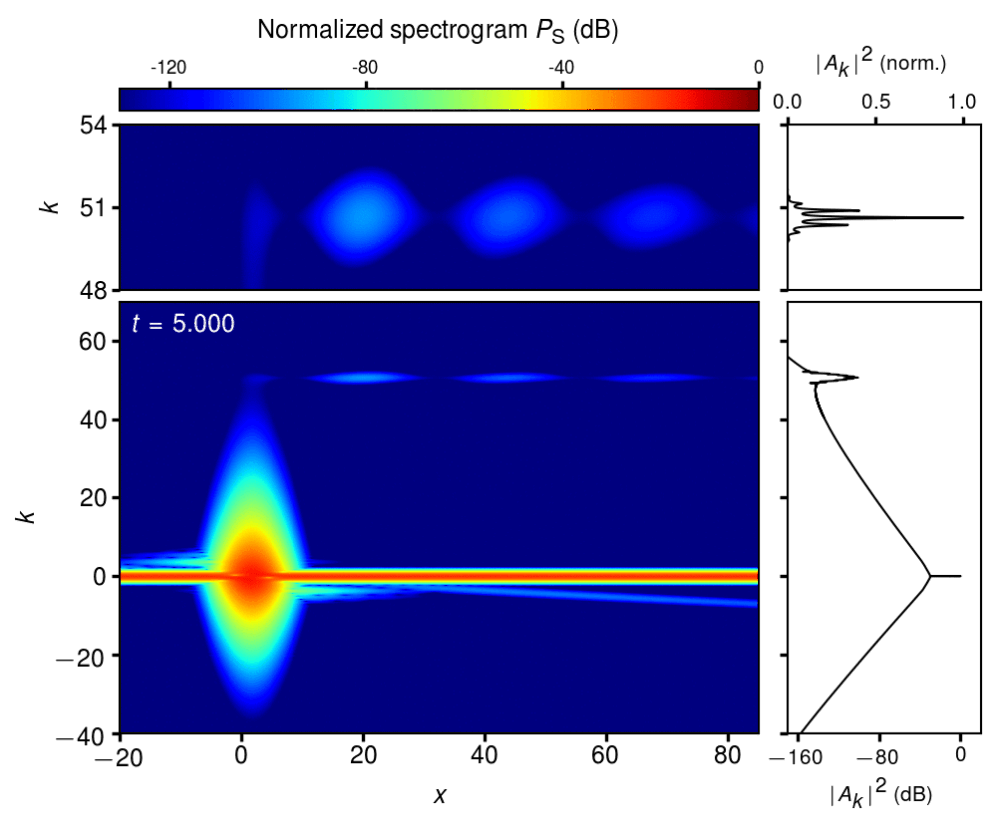

Supplement: Supplementary file 2 — Supplementary Information2. [file 41598_2020_65426_MOESM2_ESM.gif]
